# Novel therapeutic regimens against *Helicobacter pylori*: an updated systematic review

**DOI:** 10.3389/fmicb.2024.1418129

**Published:** 2024-06-07

**Authors:** Ting-Ting Huang, Yong-Xiao Cao, Lei Cao

**Affiliations:** ^1^Department of Pharmacology, School of Basic Medical Science, Xi’an Jiaotong University Health Science Center, Xi’an, Shaanxi, China; ^2^Precision Medical Institute, The Second Affiliated Hospital of Xi’an Jiaotong University, Xi’an, Shaanxi, China

**Keywords:** *Helicobacter pylori*, antibacterial resistance, probiotic, natural products, vaccine, oxygen, agonist, antagonist

## Abstract

*Helicobacter pylori* (*H. pylori*) is a strict microaerophilic bacterial species that exists in the stomach, and *H. pylori* infection is one of the most common chronic bacterial infections affecting humans. Eradicating *H. pylori* is the preferred method for the long-term prevention of complications such as chronic gastritis, peptic ulcers, gastric mucosa-associated lymphoid tissue lymphoma, and gastric cancer. However, first-line treatment with triple therapy and quadruple therapy has been unable to cope with increasing antibacterial resistance. To provide an updated review of *H. pylori* infections and antibacterial resistance, as well as related treatment options, we searched PubMed for articles published until March 2024. The key search terms were “*H. pylori*”, “*H. pylori* infection”, “*H. pylori* diseases”, “*H. pylori* eradication”, and “*H. pylori* antibacterial resistance.” Despite the use of antimicrobial agents, the annual decline in the eradication rate of *H. pylori* continues. Emerging eradication therapies, such as the development of the new strong acid blocker vonoprazan, probiotic adjuvant therapy, and *H. pylori* vaccine therapy, are exciting. However, the effectiveness of these treatments needs to be further evaluated. It is worth mentioning that the idea of altering the oxygen environment in gastric juice for *H. pylori* to not be able to survive is a hot topic that should be considered in new eradication plans. Various strategies for eradicating *H. pylori*, including antibacterials, vaccines, probiotics, and biomaterials, are continuously evolving. A novel approach involving the alteration of the oxygen concentration within the growth environment of *H. pylori* has emerged as a promising eradication strategy.

## 1 Introduction

*Helicobacter pylori* is a spiral-shaped gram-negative bacterium. Its persistent infection is associated with severe gastric complications (Malfertheiner et al., 2023) and plays a significant role in the development of conditions such as gastric cancer, duodenal ulcers, and gastric ulcers, particularly contributing to the onset of gastric adenocarcinoma (Sung et al., 2021). Accurate diagnosis of *H. pylori* infection is essential for effective treatment. diagnostic methods include non-invasive and invasive approaches (Chen Y. C. et al., 2024). Non-invasive tests comprise the ^13^C-urea breath test, fecal antigen detection, serological tests, and molecular biology tests (e.g., PCR). Invasive tests involve endoscopy with tissue biopsy, the rapid urease test, and histopathology. Patients diagnosed with *H. pylori* infection typically develop chronic gastritis, making *H. pylori* gastritis an infectious disease that warrants eradication therapy (Ranjbar et al., 2017).

Since the discovery of *H. pylori* in 1983, its eradication treatments have evolved from single antibiotics (e.g., metronidazole or amoxicillin) to combinations of multiple antibiotics. Standard triple therapies, which include a proton pump inhibitor such as omeprazole along with two antibiotics like clarithromycin and amoxicillin or metronidazole, have been superseded by quadruple therapies. These quadruple therapies involve a proton pump inhibitor, bismuth agents such as bismuth potassium citrate, and two antibiotics (Malfertheiner et al., 2024). Addressing *H. pylori* infection requires appropriate antibacterial treatment. While the incidence of *H. pylori* infection has declined in developed countries (Megraud et al., 2021), it remains widespread in developing nations, with increasing drug resistance. In most cases, the infection is acquired during childhood (Malaty et al., 2002; Park et al., 2021; Yuan et al., 2022). The prevalence rate may also be influenced by local economic conditions and health care infrastructure.

Eradicating *H. pylori* requires a suitable treatment regimen. However, treating *H. pylori* infection presents challenges. Standard triple therapy (STT) comprises a proton pump inhibitor and two antibiotics. Owing to increasing antimicrobial resistance, STT is no longer effective for treating *H. pylori* infection (Ho et al., 2022; Boyanova et al., 2023). Consequently, the search for new *H. pylori* treatments and strategies to reduce antimicrobial resistance has become a primary focus.

This study comprehensively reviews *H. pylori* infection, antimicrobial treatment approaches, drug resistance, and the development of new anti-*H. pylori* drugs or novel ideas to eradicate *H. pylori,* which introduces novel perspectives that can optimize future treatment strategies.

## 2 The prevalence of *Helicobacter pylori* infection

**Figure 1:**
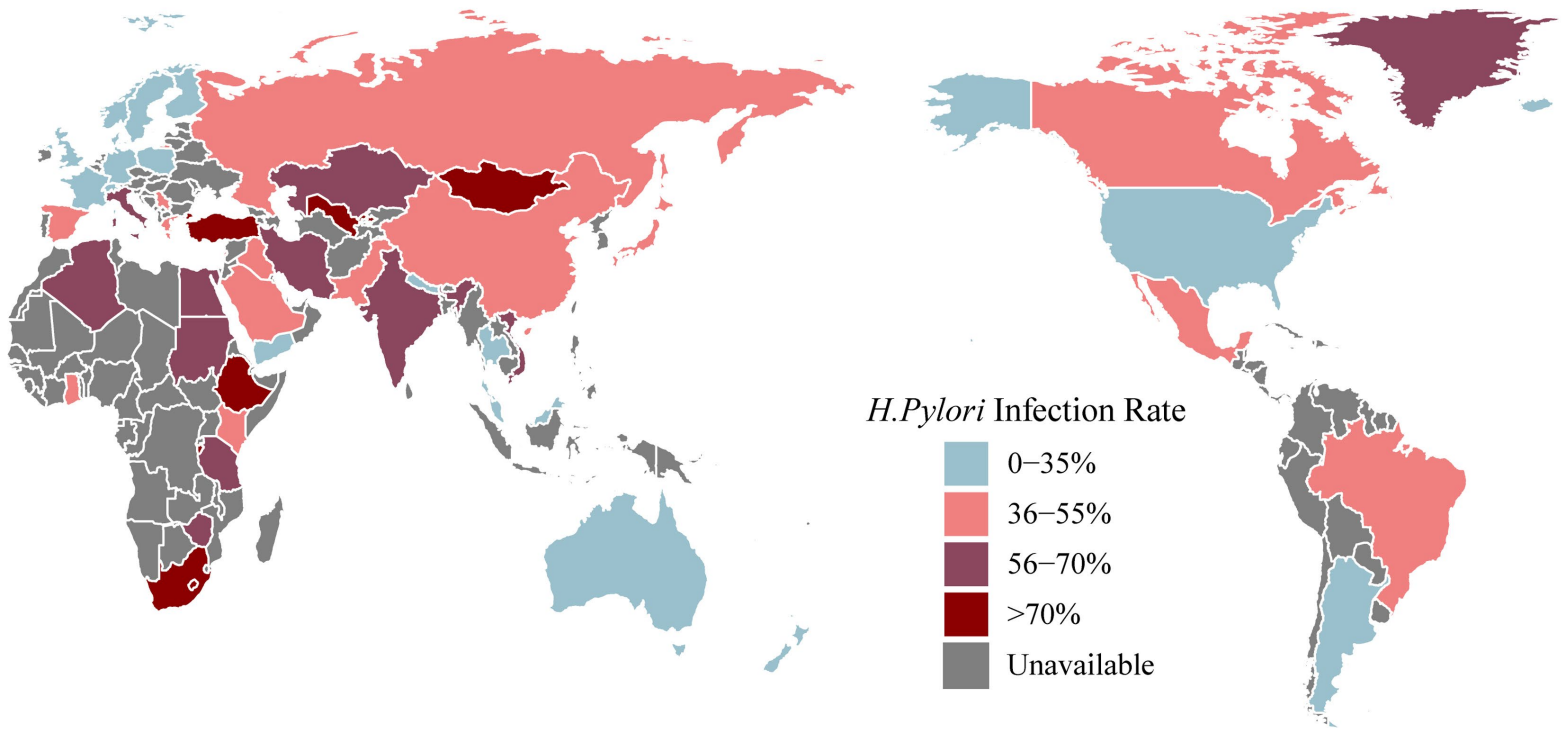
The latest prevalence of *Helicobacter pylori* infection [Antarctica removed (no data collected)].

*Helicobacter pylori* gastritis (Sugano et al., 2015) is an infectious disease that has been included in the 11th revision of the International Classification of Diseases (Malfertheiner et al., 2022). *H. pylori* infection has been widespread worldwide (Figure 1; Table 1), with an overall infection rate as high as 43.2% (95% CI: 40.3–45.9%) in 2011–2022 (Li et al., 2023). *H. pylori* infection is highly prevalent in East Asia. A systematic review and meta-analysis covering 1,377,349 individuals from 412 eligible studies indicated that the *H. pylori* infection rate in mainland China was 40% in 2015–2019 (Ren et al., 2022). The epidemiological landscape of *H. pylori* in Japan is evolving, with an infection rate of 70% in less developed areas and 40% in more developed regions (Inoue, 2017). In a Korean population of 23,770 people, the detection of anti-*H. pylori* IgG antibodies, indicating the presence of *H. pylori* infection, revealed a seroprevalence rate of 41.5% (Lim et al., 2018). A recent cross-sectional study in India, using 2,998 biopsy samples, indicated an overall prevalence of *H. pylori* infection of approximately 64% (Venkatesan et al., 2024).

**Table 1:** The primary and secondary resistance rate and mechanism of antibiotics (Savoldi et al., 2018; Boyanova et al., 2020; Gisbert, 2020; Schubert et al., 2021; Chen et al., 2022; Borka Balas et al., 2023).

Antibiotics	Primary Antibiotic Resistance % (95% CI)	Secondary Antibiotic Resistance% (95% CI)	Resistance mechanisms	Gene or sequence name
β-Lactams (e.g., amoxicillin)	EMR: 14 (8–20), AR: 10 (2–19), ER:0 (0–2), SAR: 2 (0–5), WPR: 1 (1–1)	EMR:10 (5–18), AR: 7 (1–13), Australia:2.5 (0.041–11.0) WPR: 1 (1–2)	mutations in structural alterations in penicillin-binding proteins	*pbp-1A, pbp2, pbp3, pbp4, hofH, hefC, hopC*
Fluoroquinolones (e.g., levofloxacin)	EMR:19 (10–29), AR: 15 (5–16), ER: 11 (9–13), SAR: 30 (14–46), WPR: 22 (17–28)	EMR: 30 (14–46), AR: 22 (3–42), ER: 11 (9–13), SAR: 24 (15–37), WPR:30 (20–39)	Mutations in the quinolone resistance-determining region (QRDR) of the gyrA gene encoding the A subunit of DNA gyrase	*gyrA, gyrB*
Macrolides (e.g., clarithromycin)	EMR:33 (23–44), AR: 10 (4–16), ER: 18 (16–20), SAR: 10 (5–16), WPR: 34 (30–38)	EMR: 17 (10–27), AR: 18 (13–23), ER: 48 (38–57), SAR: 15 (8–27)^a^, WPR: 67 (54–80)	Mutations in domain V of the *23S rRNA* gene	23S rRNA, *rpl22, infB*
Nitroimidazoles (e.g., metronidazole)	EMR: 56 (46–66), AR: 23 (2–44), ER: 32 (27–36), SAR: 51 (26–76), WPR: 47 (37–57)	EMR: 65 (54–74) ^a^, AR: 30 (19–41), ER: 48 (38–58), SAR: 44 (32–58) ^a^, WPR: 62 (50–71)	mutations in enzymes involved in DNA repair and in response to oxidative stress, extensive mutational changes in the gene encoding *RdxA,*	*rdxA-related and rdxA-related promoter region, frxA, fur sodB-related promoter region, recA, mdaB, ribF, omp11,rpsU*
Tetracyclines (e.g., tetracycline)	EMR: 10 (4–15), WPR: 2 (1–2), China: 2 (1–4), in most countries worldwide ≤10%	EMR: 17 (8–26), Australia: 0.53 (0.001–2.21), WPR: 0 (0–1),	*16S rRNA* with a tetracycline-binding pocket altered by single, double or triple base-pair substitutions	*16S rRNA*
Rifamycins (e.g., rifabutin)	France: 0.7 (0.1–2.7), Overall 0.07	France: 0.9 (0–4.7)	mutations in DNA-directed RNA polymerase, mostly the β-subunit encoded by the *rpoB* gene	*rpoB*
Nitrofurans (e.g., furazolidone)	China:1 (0–4), Australasia:3.72 (0.0038–14.7)	Australia: 8.36 (3.4–15.3)	mutations in *PorD* and/or *OorD* with missense	*porD, oorD*

EMR, Eastern Mediterranean region; AR, American region. ER, European region; SAR, Southeast Asia region; WPR, Western Pacific region.^1^Only one study contributed to analysis.

In North America, the prevalence of *H. pylori* infection from 1980 to 2022 was 36.2% (95% CI: 22.6–49.9%) (Li et al., 2023). A meta-analysis covering the years 1999–2018 reported a diagnosis rate of *H. pylori* infection of 25.8% in the USA (Shah et al., 2024). Willems et al. reported that the *H. pylori* infection rate in Canada was approximately 20–30% (Willems et al., 2020). Victor et al. collected dental plaque samples from 36 families in a southwestern Mexican community and reported an *H. pylori* infection rate of 41.7% (Urrutia-Baca et al., 2023). In a urease test report from Brazil, involving 852 individuals, the overall *H. pylori* infection rate was 35.4%, with a specific infection rate of 24.7% among children and adolescents (Toscano et al., 2018). The *H. pylori* infection rate in Argentina falls between the high incidence observed in African countries and the lower incidence observed in other countries in the Americas (Olmos et al., 2000).

The overall prevalence of *H. pylori* infection in Europe was reported to be 47.5% (95% CI: 43.0–52.1%) (Li et al., 2023). Bordin compared *H. pylori* infection rates across all Russian Federation communities in 2017 and 2019. Among untreated persons, the overall *H. pylori* infection rates were 41.8% in 2017 and 36.4% in 2019 (Bordin et al., 2022). In a seven-year prospective cross-sectional study examining the presence of *H. pylori* infection among 3,241 students in Gruzice, Poland, the positivity rate was 23.6% (Szaflarska-Poplawska and Soroczynska-Wrzyszcz, 2019). The reported prevalence of *H. pylori* infection in Germany in 2018 ranged from 20 to 40% (Fischbach and Malfertheiner, 2018). A recent meta-analysis reported infection rates of 28.6% in France, 18.8% in Denmark and 26.3% in the United Kingdom (Chen Y. C. et al., 2024).

In the representative countries of Oceania, namely, Australia and New Zealand, *H. pylori* infection rates vary. As reported in a global meta-analysis from 2010 to 2022, Australia recorded a rate of 24.8%, while New Zealand had a rate of 9.2% (Chen Y. C. et al., 2024). Biopsy samples collected from 12,482 individuals in South Australia from 1998 to 2017 revealed a positive *H. pylori* infection rate of 11.5% (Schubert et al., 2022).

The *H. pylori* infection rate in Africa was reported to be 52.6% (Chen Y. C. et al., 2024). Notably, the prevalence in Africa surpasses that in other continents and exhibits significant variability, depending on the geographical location. In a comprehensive review of *H. pylori* infection in the Middle East and North Africa, infection rates displayed wide-ranging disparities within regions, ranging from 7 to 50% in young children and from 36.8 to 94% in adults (Alsulaimany et al., 2020). A review by Smith revealed elevated infection rates, reaching 87.8% in northern Nigeria and 70.8% in Burundi and exceeding 80% in Ethiopia (Smith et al., 2022). A 2019 report from Egypt revealed an overall *H. pylori* infection rate of 66.1% (Galal et al., 2019).

## 3 *Helicobacter pylori* infectious diseases and its morphological form

**Figure 2:**
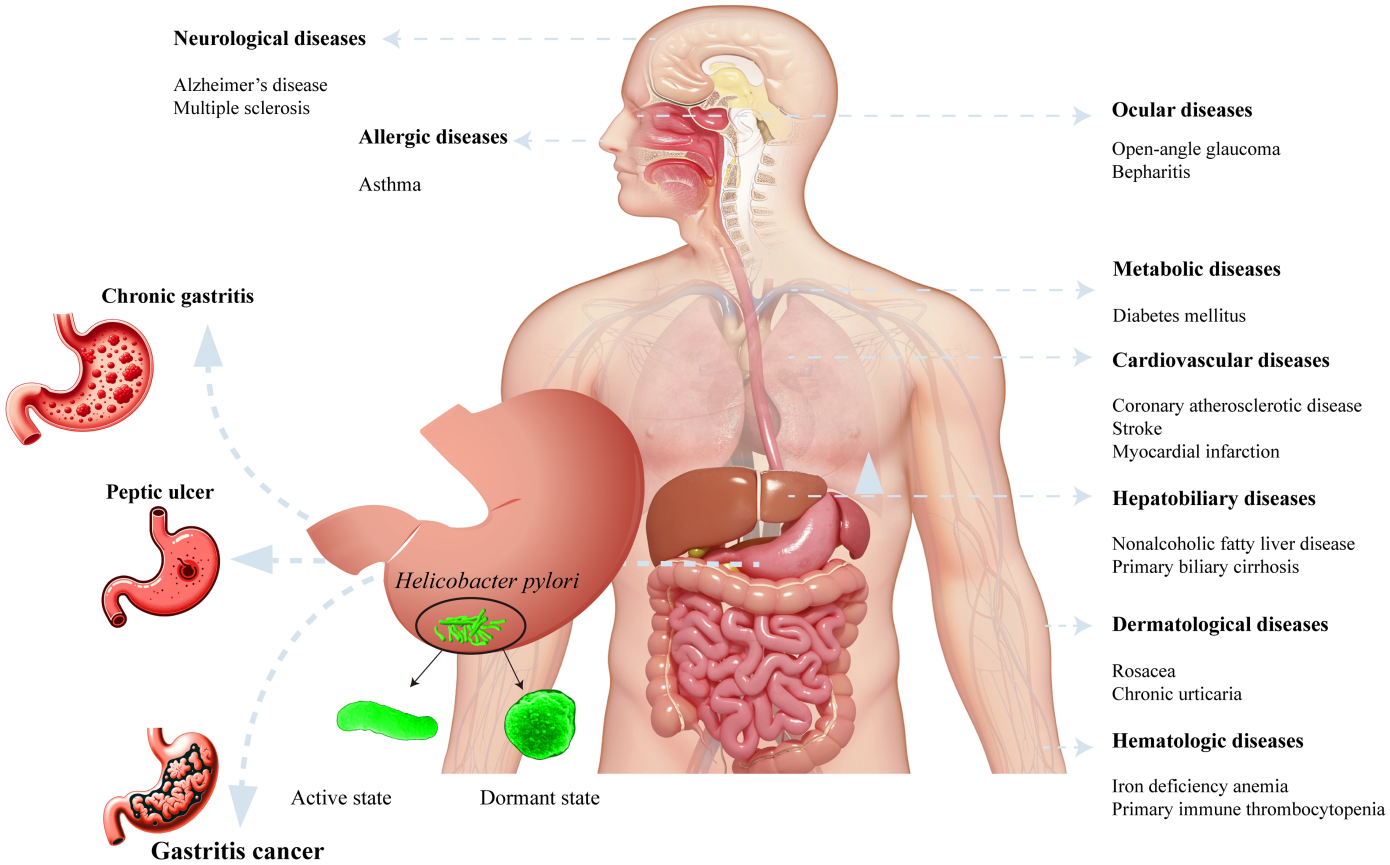
*Helicobacter pylori* infection associated diseases and its morphological form.

After *H. pylori* infection, various gastrointestinal and systemic diseases ensue in the human body (Figure 2). *H. pylori* infection always causes chronic gastritis, and approximately 10–15% of infected individuals may develop peptic ulcers. Peptic ulcer disease has a lifetime risk of approximately 17% and may lead to serious complications, such as bleeding, perforation, and gastric cancer. Chronic atrophic gastritis is currently considered to be a precancerous lesion.

An increasing focus has been directed toward clinical extragastric manifestations following *H. pylori* infection. Research exploring the relationship between *H. pylori* infection and cardiovascular-related diseases has gained substantial attention. The presence of *H. pylori* infection in patients increases susceptibility to coronary atherosclerotic disease (Chen et al., 2013; Wang et al., 2021), stroke (Sun et al., 2023), and myocardial infarction (Wang et al., 2023). Moreover, *H. pylori* infection has been linked to neurological disorders, including Alzheimer’s disease (Kandpal et al., 2024) and multiple sclerosis (Arjmandi et al., 2022). Eye-related ailments such as open-angle glaucoma (Doulberis et al., 2020) and blepharitis (Sacca et al., 2006) may also result from infection. In children and young individuals, *H. pylori* infection has been associated with the onset of allergic systemic conditions like Asthma (Durazzo et al., 2021). *H. pylori* infection can induce alterations in the metabolic system, contributing to blood sugar disorders in individuals with diabetes (Chang et al., 2023). Additionally, stomach colonization by *H. pylori* has been implicated in accelerating liver fibrosis (Okushin et al., 2018) and mediating tumorigenesis. Encouragingly, the eradication of *H. pylori* has led to improvements in hematologic disorders, including iron deficiency anemia (Elbehiry et al., 2023) and primary immune thrombocytopenia (Wang et al., 2022).

The morphological form of *H. pylori* plays a pivotal role in disease development among *H. pylori*-infected patients. In human gastric biopsy specimens, *H. pylori* predominantly exists in a spiral form (Gladyshev et al., 2020). Under adverse conditions, such as high temperature, pH fluctuations or the use of antibacterial drugs, *H. pylori* can transform into a latent spherical form. The intermediate stages between the spiral and spherical forms are referred to as the C-shaped and/or U-shaped forms of *H. pylori*, also known as the dormant state. Dormancy is regarded as a reversible state in which bacterial cells exhibit reduced metabolic activity. When favorable culture conditions are restored, the cells regain metabolic activity and can be cultured (Ierardi et al., 2020). Various antibiotics (amoxicillin, clarithromycin, metronidazole, and tetracycline) at the minimum inhibitory concentrations (MICs) induce a spherical form. Furthermore, after exposing *H. pylori* to omeprazole (a proton pump inhibitor) at 0.5 MIC for 3, 6, and 9 days, the bacterium could revert to the spiral form after omeprazole removal from the culture medium (Krzyzek and Grande, 2020). Patients with *H. pylori* infection is susceptible to reinfection if the bacterium is not completely eradicated. Vasiliy et al. noted that after one year of anti-*H. pylori* treatment in patients with duodenal peptic ulcers, the identification of *H. pylori* suggested that the bacterium may have transitioned from the dormant state to an active state (Reshetnyak and Reshetnyak, 2017). Nikita et al. reported that the virulence of the spherical form was comparable to that of the spiral form and that the spherical form increased the risk of developing gastric cancer in patients (Gladyshev et al., 2020). In summary, when patients with *H. pylori* infection exhibit significant symptoms, such as peptic ulcers, eradication therapy should be initiated. However, incomplete eradication with antibiotics and proton pump inhibitors may lead to the conversion of the spherical form back to the active spiral form.

## 4 *Helicobacter pylori* colonization and its virulence mechanisms

*Helicobacter pylori* colonizes the gastric mucosa and is highly adapted to the acidic environment through complex molecular processes. Adhesion mechanisms are crucial for its sustained colonization. Two key outer membrane proteins, blood group antigen-binding adhesion (BabA) and salivary acid-binding adhesion (SabA), play significant roles (Malfertheiner et al., 2023). BabA, one of the earliest discovered *H. pylori* adhesins, recognizes and binds to various antigens, particularly the ABO group, facilitating survival and colonization in the stomach (Chang et al., 2023). SabA, also known as HopP, binds to sialylated Lewis x (sLex) antigens on gastric epithelial cells, promoting adhesion and survival despite the host’s immune response (Pang et al., 2014; Doohan et al., 2021). BabA initiates adhesion by binding to antigens, while SabA maintains persistent infection in an inflammatory environment. Their synergistic action and dynamic regulation enable *H. pylori* to efficiently colonize the host and cause disease.

Inflammasomes play a crucial role in the development of gastric inflammation caused by *H. pylori* (Kandpal et al., 2024). These important cytoplasmic multiprotein complexes, when activated, promote the release of pro-inflammatory cytokines such as IL-1β and IL-18 (Zheng et al., 2024). Among the various inflammasomes, the NLRP3 inflammasome is the most commonly studied in *H. pylori* infection. When exposed to intracellular stimuli, such as bacterial toxins and oxidative stress, or danger signaling molecules, including the loss of intracellular potassium ions and pH alterations (Mugengana et al., 2021), NLRP3 proteins are activated. This activation accelerates the development of peptic ulcers associated with *H. pylori* infection.

The primary virulence factors of *H. pylori* include Cytotoxin-associated gene A (*CagA*), Vacuolating cytotoxin A (*VacA*), Duodenal ulcer-promoting gene A (*DupA*), and Urease (Malfertheiner et al., 2023). *CagA* is a pathogenic protein delivered into host cells via the Type IV secretion system (T4SS) (Bhattacharjee et al., 2024). It activates multiple signaling pathways, such as NF-κB, MAPK/PI3K, and Wnt/β-catenin, affecting cell proliferation, motility, polarity, apoptosis, and inflammation (Ayana et al., 2014; Hayashi et al., 2017). VacA forms an anion-selective membrane channel upon contact with host cells, causing the formation of large intracellular vacuoles (Yahiro et al., 2015; Nejati et al., 2018), disrupting mitochondrial function, inducing apoptosis and autophagy, and inhibiting T and B cell proliferation (Cover and Blanke, 2005). DupA, associated with T4SS-related proteins, is implicated in urease regulation (Asimellis et al., 2005), ATPase-associated efflux pumps, and apoptotic pathways, potentially enhancing *H. pylori* virulence (Chen et al., 2018). Urease, composed mainly of the ureA and ureB genes and cofactors, including nickel, and regulated by the gastric microenvironment’s pH, hydrolyzes urea into ammonia and carbon dioxide. This reaction neutralizes gastric acid, creating a protective microenvironment that promotes colonization and evades the host immune system (Ha et al., 2001; Allen et al., 2023; Malfertheiner et al., 2023).

## 5 Antibacterial treatment of *Helicobacter pylori* infection

**Figure 3:**
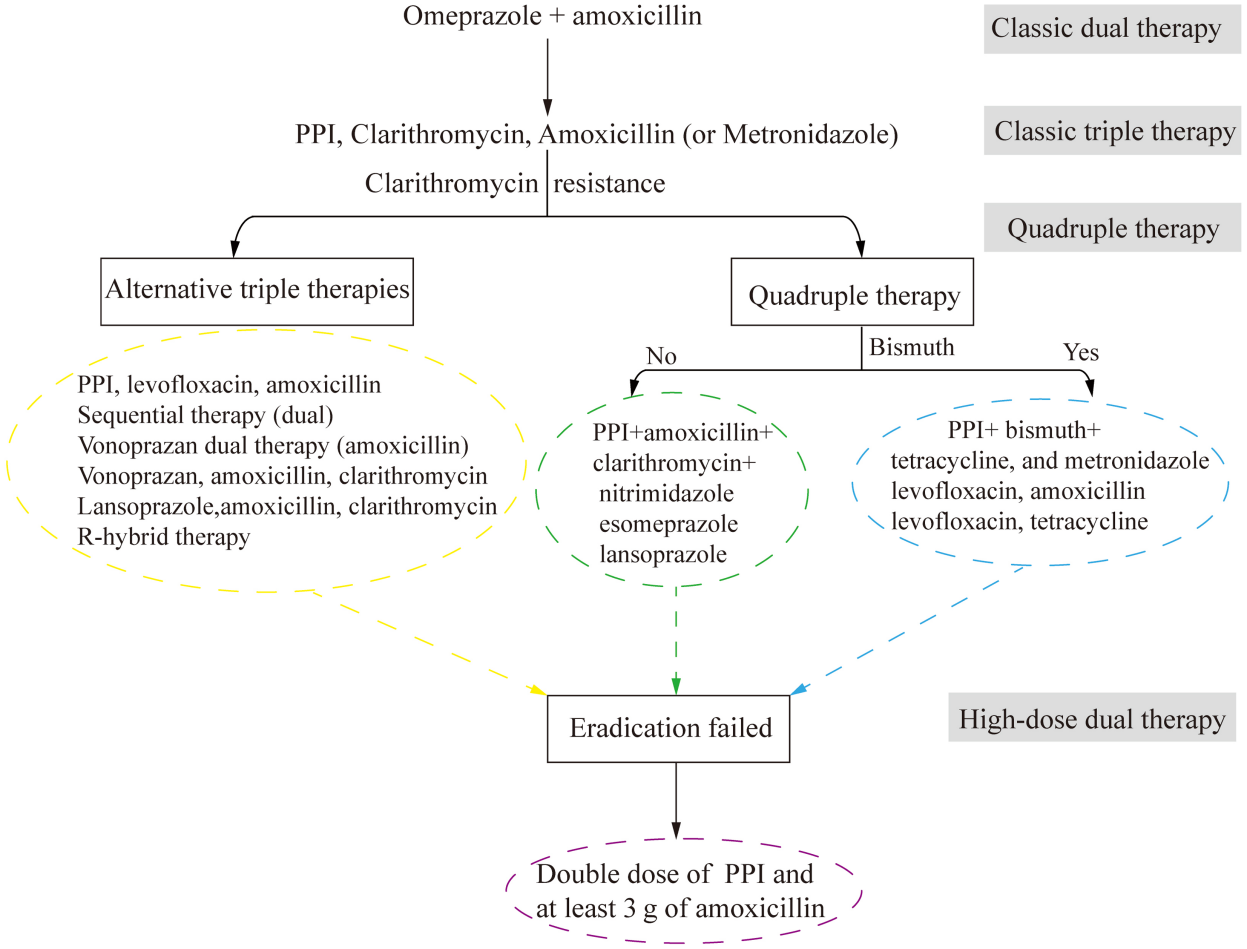
The development of antimicrobial treatment for *H. pylori* infection. PPI, proton pump inhibitors.

Advancements in the understanding of *H. pylori* infection have transformed peptic ulcer treatment from anti-acid therapies to antibacterial-based approaches (Figure 3). The eradication of *H. pylori* by antibacterial drugs promotes ulcer healing and reduces recurrence rates. In the late 20th century, antibacterial drugs were less effective in the acidic environment of the stomach. Therefore, treatments targeting both bacteria and stomach acid were needed. Omeprazole and amoxicillin were used to be a classical dual therapy, which was highly effective and well tolerated owing to its bactericidal and antacid effects (Labenz et al., 1994). Nevertheless, plans to eradicate *H. pylori* have shifted to triple therapy, mainly including bismuth +2 antibacterial drugs or proton pump inhibitors (PPIs) + 2 antibacterial drugs (consisting of amoxicillin and clarithromycin, metronidazole or levofloxacin).

Increasing antibacterial resistance leads to a further reduction in treatment efficacy. For example, a successful 14-day concomitant therapy containing 1 g of metronidazole and clarithromycin produced 14,000 kg of unnecessary antibacterial agents per 1 million successful treatments (Graham, 2020). To address this issue, alternative therapies, such as Vono-triple therapy (a vonoprazan-containing therapy) and R-hybrid therapy (a reverse hybrid therapy), have been developed and have achieved eradication rates greater than 90% those of STT. However, while R-hybrid therapy has a high cure rate, it has failed to achieve significant comparative efficacy. One promising drug is vonoprazan, a potassium-competitive acid blocker (Rokkas et al., 2021).

In areas with high clarithromycin resistance, STT has been replaced with bismuth-containing quadruple therapy (BQT). The improved bismuth-containing quadruple therapy (mBCQT) consists of a PPI, bismuth, metronidazole, and tetracycline. The results showed that mBCQT achieved an *H. pylori* eradication rate of 88.2% after continuous treatment of patients for two weeks (Kim et al., 2019). In Greece, where clarithromycin resistance is greater than 20% and bismuth is not available, a 10-day nonbismuth-based quadruple concomitant therapy (esomeprazole, clarithromycin, amoxicillin, and metronidazole) is currently the first-line treatment for *H. pylori* eradication (Apostolopoulos et al., 2020). Hybrid therapy, which combines sequential therapy and concomitant therapy, is a novel two-step therapy (Chey et al., 2018). A 14-day hybrid therapy regimen (pantoprazole plus amoxicillin for 7 days, followed by pantoprazole plus amoxicillin, clarithromycin, and metronidazole for another 7 days) had an *H. pylori* eradication rate of 93.9% (154/164; 95% confidence interval [CI]: 90.3 to 97.5%). The *H. pylori* eradication rate of bismuth quadruple therapy (pantoprazole, bismuth subcitrate, tetracycline, and metronidazole for 14 days) was 92.8% (154/166; 95% CI: 88.9 to 96.7%). The *H. pylori* eradication rates of the two therapies are similar, but hybrid therapy has fewer adverse effects than bismuth quadruple therapy (Tsay et al., 2017). For patients allergic to penicillin who are infected with *H. pylori* for the first time, the triple therapy of bismuth (using bismuth potassium citrate and esomeprazole) with minocycline, cefuroxime, and full-dose metronidazole (pairwise) has the same satisfactory efficacy in *H. pylori* eradication, with a good safety and compliance (Zhang Y. X. et al., 2023). Nonbismuth or bismuth quadruple therapy is characterized by poor compliance and a high cost, similar to many other drugs.

High-dose dual therapy (HDDT) consisting of a PPI and amoxicillin has been proven to have a better *H. pylori* eradication rate and fewer adverse reactions than traditional triple or quadruple therapy (Yang et al., 2019; Zhu et al., 2020). The HDDT group (40 mg of esomeprazole and 100 mg of amoxicillin three times daily) and the bismuth-containing quadruple therapy group, which was called the TFEB group (40 mg of esomeprazole, 220 mg of bismuth potassium citrate, and 100 mg of furazolidone twice daily, combined with 500 mg of tetracycline three times daily), were treated continuously for 14 days, and the eradication rates of *H. pylori* reinfection in patients who failed eradication were similar in both groups. However, the incidence of adverse events in the HDDT group was significantly lower than that in the TFEB group (11.1% vs. 26.8%, *p* < 0.001), and the treatment compliance was good (Bi et al., 2022). The replacement of PPIs with vonoprazan was also proven to be effective. After 10 days of continuous administration, the eradication rates were 93.4% for VHA-dual therapy (20 mg of vonoprazan twice daily +750 mg of amoxicillin four times daily) and 90.9% for B-quadruple therapy (20 mg of esomeprazole +200 mg of bismuth +1,000 mg of amoxicillin +500 mg of clarithromycin twice daily). Although compliance was similar between the two therapies, the former had a lower rate of adverse events (Qian et al., 2023). VHA-dual therapy is expected to become the first-line therapy for *H. pylori* eradication.

In addition to the use of modifying drugs to improve eradication rates, patient compliance is also a factor that directly affects treatment success. Patients received telephone follow-ups on the 3rd, 14th, and 30th days of treatment, and the results showed that semiautomatic intensive follow-ups improved the *H. pylori* eradication rate and patient compliance (Chen et al., 2021). The use of technology-enhanced communication strategies, such as telephone re-education, text messaging, social media re-education, and structured patient counseling programs, enables patients to comprehensively understand the treatment process, leading to improved eradication rates and compliance (Zhao et al., 2020; Chua et al., 2022).

## 6 The resistance of *Helicobacter pylori* to antibacterial drugs

*Helicobacter pylori* colonizes the surface of the gastric mucosa and is protected by the gastric mucus layer, making it difficult to remove *H. pylori* from the stomach. Resistance of *H. pylori* gradually develops over the course of treatment owing to limited selection of antibacterial agents and the limited frequency of treatment. Furthermore, when patients who have previously used antibacterial drugs are treated, the resistance rate increases, as does the risk of treatment failure (Guo C. G. et al., 2022). Clarithromycin is one of the most important drugs for the treatment of *H. pylori* worldwide, but its primary resistance rate has significantly increased, from 17.2 to 19.7% or even 27.2% (Kasahun et al., 2020). Metronidazole shows a universal drug resistance pattern worldwide, with the resistance rate > 15% on all continents (Table 1; Savoldi et al., 2018; Boyanova et al., 2020; Gisbert, 2020; Schubert et al., 2021; Chen et al., 2022; Borka Balas et al., 2023). The primary resistance rates are expected to continue increasing in the future (Savoldi et al., 2018). Resistance rates are on the rise, affecting not only clarithromycin and metronidazole but also drugs such as levofloxacin, which are often deemed alternative therapies. Currently, the rates of *H. pylori* resistance to clarithromycin, metronidazole, and levofloxacin are 27.2, 39.7, and 22.5%, respectively, worldwide (Kasahun et al., 2020). In the United States, the rates of resistance to clarithromycin, metronidazole, and levofloxacin are 17.6, 43.6, and 57.8%, respectively (Hulten et al., 2021). Over a 10-year period, the resistance rate to clarithromycin increased from 17.5 to 21.4% in European countries. The *H. pylori* resistance was assessed, and 201 strains showed double resistance, most commonly to clarithromycin and metronidazole (Megraud et al., 2021). In the Asia Pacific region, overall resistance to clarithromycin increased from 7 to 21% over the same period (Jiang et al., 2022). In a study of 125 Vietnamese patients with peptic gastric ulcers, the percentages of patients who were resistant to amoxicillin, clarithromycin, metronidazole, levofloxacin, and tetracycline were 27.5, 50.0, 67.5, 35.0, and 5.0%, respectively. Additionally, 7.5% of the patients were sensitive to all five antibacterial drugs, 27.5% were single-drug resistant, 42.5% were double-drug resistant, 17.5% were triple-drug resistant, and 5% were quadruple-drug resistant (Vu et al., 2022).

Primary resistance rates for clarithromycin, metronidazole, levofloxacin, and amoxicillin vary across different regions (Table 2). Metronidazole generally showed higher resistance rates than the other three antibiotics, with rates reaching up to 91% in Africa and the lowest at approximately 25% in Europe. Clarithromycin also exhibited substantial resistance, peaking at around 37% in Asia. Resistance rates for levofloxacin were highest in Asia at about 25%, with other regions not exceeding 20%. Amoxicillin consistently had low resistance rates, maintaining around 1% in Europe.

**Table 2:** Primary *H. pylori* resistance in Asia, Africa, Europe, and America.

Drug	Region	Data collection Year	Prevalence	References
Clarithromycin	Asia	2001 ~ 2022	37.0%	Zhou et al. (2024)
		1990 ~ 2022	22.0%	Hong et al. (2024)
		~2022	27.0%	Shrestha et al. (2023)
	Africa	2007 ~ 2017	15.0%	Borka Balas et al. (2023)
		2015 ~ 2016	29.2%	Fauzia et al. (2020)
		2009 ~ 2014	5.5%	Ghotaslou et al. (2015)
	Europe	2009 ~ 2014	22.1%	Ghotaslou et al. (2015)
		2013 ~ 2021	22.0%	Bujanda et al. (2024)
		2017 ~ 2020	17.7%	Le Thi et al. (2023)
		2013 ~ 2020	25.0%	Bujanda et al. (2024)
	America	2007 ~ 2017	10.0%	Borka Balas et al. (2023)
		2011 ~ 2021	31.5%	Ho et al. (2022)
		2006 ~ 2016	10.0%	Savoldi et al. (2018)
Metronidazole	Asia	2001 ~ 2022	51.0%	Chen P. et al. (2024)
		1990 ~ 2022	52.0%	Hong et al. (2024)
		~2022	69.0%	Shrestha et al. (2023)
	Africa	2007 ~ 2017	91.0%	Borka Balas et al. (2023)
		2015 ~ 2016	75.8%	Fauzia et al. (2020)
		2009 ~ 2014	75.0%	Ghotaslou et al. (2015)
	Europe	2009 ~ 2014	31.2%	Ghotaslou et al. (2015)
		2013 ~ 2021	27.0%	Bujanda et al. (2024)
		2017 ~ 2020	25.0%	Le Thi et al. (2023)
		2013 ~ 2020	30.0%	Bujanda et al. (2024)
	America	2007 ~ 2017	23.0%	Borka Balas et al. (2023)
		2011 ~ 2021	42.1%	Ho et al. (2022)
		2006 ~ 2016	23.0%	Savoldi et al. (2018)
Levofloxacin	Asia	2001 ~ 2022	19.0%	Chen P. et al. (2024)
		1990 ~ 2022	26.0%	Hong et al. (2024)
		~2022	34.0%	Shrestha et al. (2023)
	Africa	2007 ~ 2017	14.0%	Borka Balas et al. (2023)
		2009 ~ 2014	15.0%	Ghotaslou et al. (2015)
	Europe	2009 ~ 2014	14.2%	Ghotaslou et al. (2015)
		2013 ~ 2021	18.0%	Bujanda et al. (2024)
		2013 ~ 2020	20.0%	Bujanda et al. (2024)
	America	2007 ~ 2017	15.0%	Borka Balas et al. (2023)
		2011 ~ 2021	37.6%	Ho et al. (2022)
		2006 ~ 2016	15.0%	Savoldi et al. (2018)
Amoxicillin	Asia	2001 ~ 2022	3.0%	Chen P. et al. (2024)
		1990 ~ 2022	4.0%	Hong et al. (2024)
		~2022	23.0%	Shrestha et al. (2023)
	Africa	2007 ~ 2017	38.0%	Borka Balas et al. (2023)
		2015 ~ 2016	72.6%	Fauzia et al. (2020)
		2009 ~ 2014	40.9%	Ghotaslou et al. (2015)
	Europe	2009 ~ 2014	0.4%	Ghotaslou et al. (2015)
		2013 ~ 2021	<1.0%	Bujanda et al. (2024)
		2017 ~ 2020	6.0%	Le Thi et al. (2023)
		2013 ~ 2020	0.4%	Bujanda et al. (2024)
	America	2007 ~ 2017	10.0%	Borka Balas et al. (2023)
		2011 ~ 2021	2.6%	Ho et al. (2022)
		2006 ~ 2016	10.0%	Savoldi et al. (2018)

**Figure 4:**
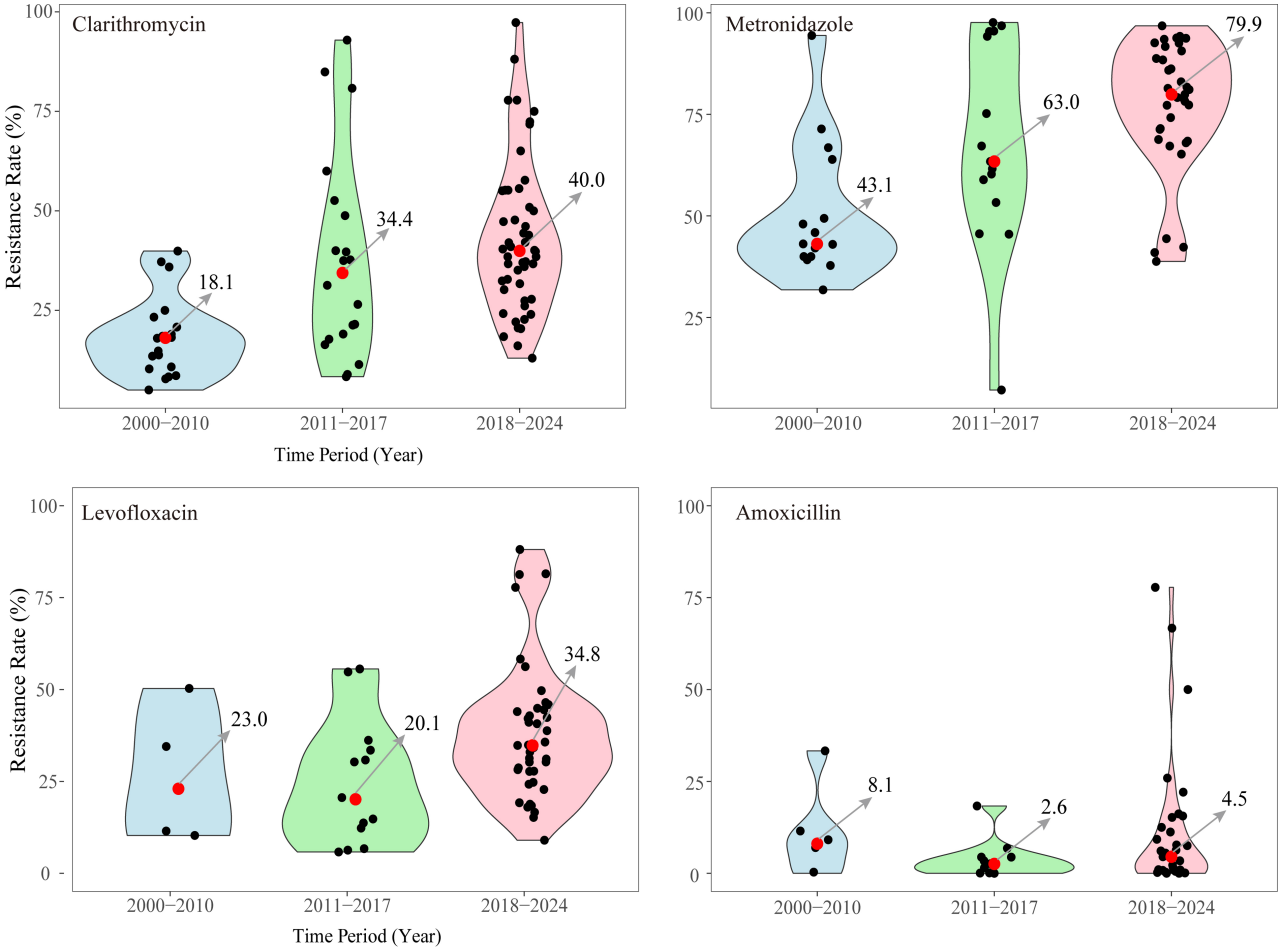
Primary resistance to 4 major antibacterial used of *H. pylori* infection in China from 2000 to 2024.

Analysis of data from China revealed that the average resistance rate to clarithromycin was 24.0%, with single-, double-, triple-, and quadruple-drug resistance rates of 54.6, 29.0, 11.7, and 0.1%, respectively (Zhang et al., 2021). In the last 20 years, primary resistance to major antibacterial drugs (clarithromycin, metronidazole, levofloxacin, and amoxicillin) has been analyzed in China (Figure 4; Tables 1–3). Clarithromycin and metronidazole, which are commonly used as first-line treatments, have shown increased resistance, with the median rates having increased from 18.1 to 40.0% and from 43.1 to 79.9%, respectively. Since 2010, levofloxacin has been increasingly used as an alternative antibiotic. A trend toward resistance has emerged, with the primary resistance at 34.8% from 2018 to 2024. Recently, high-dose amoxicillin therapy has gained popularity as a novel treatment approach. While the median resistance rate to amoxicillin remains low at 5%, the variability in the data indicates that certain regions already exhibit relatively high resistance levels. The increasing trend of amoxicillin resistance appears to be unstoppable.

**Table 3:** Development status of current vaccine candidates.

Candidate Name/Identifier^1^	Trial status	Type of vaccine	Target antigen(s)	Ref.
*Recombinant UreB/LTB* fusion Subunit vaccine	Phase III	Subunit vaccine	Serum IgG and salivary IgA	Park et al. (2006)
*EpiVax/H. pylori* vaccine	Preclinical	Epitope vaccine	IFN-γ, IL-1β, and IL-10	Moss et al. (2011)
Imevax/IMX101	Phase I	Inactivated whole cell vaccine/ Subunit vaccine	HpaA, UreB and FlaA	Oertli et al. (2013)
China Pharmaceutical University/Probiotic vaccine delivery	Preclinical	Vector vaccine	cholera toxin B subunit/ UreA	Li et al. (2014)
Wuhu Kangwei Biological technology UreB/LTB fusion vaccine	Phase III, disconnected	Subunit vaccine	UreB/LTB	Zeng et al. (2015)
Sichuan University/Urease epitope vaccine	Preclinical	Epitope vaccine	Urease	Yang et al. (2015)
Southern Medical University/Lp220 vaccine	Preclinical	Epitope vaccine	Th1-biased Response	Li et al. (2016)
Helicovaxor®	Preclinical	Epitope vaccine	Th1-biased Response	Tobias et al. (2017)
*Recombinant VacA-CagA-NAP* Subunit vaccine	Phase II/ III	Subunit vaccine	*VacA, CagA* and *NAP*	Keikha et al. (2019)
*H. pylori* immunogen-derived peptide antigen with the sequence Met-Val-Thr-Leu-Ile-Asn-Asn-Glu (MVTLINNE)	Preclinical	Nucleic acid vaccine	IgG, IgA	Espinosa-Ramos et al. (2019)
Outer membrane proteins (OMPs) and Whole cell vaccine (WCV)	Preclinical	Subunit vaccine and whole cell vaccine	OMV	Song et al. (2020)
*L. monocytogenes*-based vaccine, a multi-epitope chimeric antigen (MECU) containing multiple B cell epitopes	Preclinical	Epitope vaccine	a multi-epitope chimeric antigen	Chen X. et al. (2023)
Novel LeoA-DNA Vaccinate	Preclinical	Nucleic acid vaccine	LeoA，CD^3+^ T-cell response	Chaleshtori et al. (2024)
pIRES2-EGFP/CTB-UreI vaccinate	Preclinical	Vector vaccine	cholera toxin B subunit	Ghasemifar et al. (2024)
*Recombinant L. lactis* vaccine LL-plSAM-WAE	Preclinical	Epitope vaccine	Urease, NAP, HSP60, and HpaA	Chen P. et al. (2024)
Oral administration of DNA alginate nanovaccine	Preclinical	Nucleic acid vaccine	UreH, systemic Th1 response	Kaveh-Samani et al. (2024)

^1^This list is not exhaustive and does not encompass all recent studies on vaccines.

In recent years, antibacterial resistance in *H. pylori*-infected children has become a growing concern. Although the triple combination of PPI-CA (proton pump inhibitor, clarithromycin, and amoxicillin) is the preferred regimen, the eradication rates in children are still lower than those in adults owing to differences in susceptibility to and compliance with antimicrobial agents (Lai and Lai, 2022). Among Asian children, the primary resistance rate to clarithromycin in Southeast Asia is 10%, which is the same as that in the Americas but lower than that in Europe and the Eastern Mediterranean, where the resistance rate exceeds 15%. The primary resistance rates to metronidazole are 51% in Asia, 23% in the Americas, 91% in Africa, and 32% in Europe. As an alternative salvage therapy, levofloxacin has a primary resistance rate as high as 30% in Asia (Borka Balas et al., 2023). In Spain, 67.5% of 80 pediatric patients showed resistance to more than two antibacterial drugs, and 6.3% had double-drug resistance, with resistance to clarithromycin, metronidazole, levofloxacin, and amoxicillin being the main manifestations (Botija et al., 2021). In Germany, multidrug resistance in pediatric patients reaches 16% (Helmbold et al., 2022), and the rate of amoxicillin resistance in patients is as high as 20%, which is of great concern. In general, the drug resistance of *H. pylori* in children cannot be ignored. Owing to the low compliance of children, the eradication rate is greatly affected, and the possibility of developing multidrug resistance is greatly increased.

The effects of *H. pylori* eradication in older patients should also be considered. In a study of 264 patients diagnosed with *H. pylori* infection, the eradication rate in patients aged 40 years and older was greater than that in patients under 40 years of age (85.7% versus 54.7%, *p* = 0.002), and the eradication rate in patients older than 60 years was 100% (Tang et al., 2020). The eradication rate of the PPI-clarithromycin-amoxicillin regimen in the young and middle-aged group (≤50 years) was significantly lower than that in the aged group (>50 years) (Nishizawa et al., 2017). One possible reason for this is that younger patients have earlier exposure to clarithromycin and greater resistance, which may reduce the eradication rate. The success rates of esomeprazole treatment were significantly greater in patients over 65 years of age than in those under 65 years of age. Nonetheless, there was no significant difference in the clarithromycin resistance rates between the two groups receiving the new first-line triple therapy with vonoprazan (Saito et al., 2019), possibly due to the potent gastric acid inhibition by vonoprazan. However, another study from Japan showed that the eradication effectiveness of first-line triple therapy with amoxicillin, clarithromycin, and vonoprazan decreased with age, possibly due to reduced gastric acid secretion in older patients, reducing the need for maintenance therapy with vonoprazan (Kusunoki et al., 2019). In conclusion, the impact of age on *H. pylori* eradication is manifested through two primary mechanisms, namely, gastric acid secretion and premature exposure to antibiotic therapy. With an increasing age, there is a reduction in gastric acid secretion (Haruma et al., 2000). However, it is noteworthy that the predominant agents employed in the current first-line *H. pylori* treatment regimens often include PPIs. These agents exert a bactericidal effect by elevating the pH of gastric juice and synergizing with antibiotics (Scott et al., 1998).

In the older population, a frequent occurrence is an elevated incidence of gastric mucosal atrophy, which is coupled with diminished gastric acid secretion. Consequently, potent gastric acid inhibitors aimed at suppressing acid production are urgently needed. This context potentially elucidates the lack of discernible distinction between the efficacy of the novel gastric acid blocker vonoprazan and that of established agents, such as clarithromycin, metronidazole, or esomeprazole. Furthermore, it is important to highlight that insufficient eradication outcomes in young patients extend beyond merely premature antibiotic exposure. These suboptimal results are also intertwined with their personal habits and medication adherence (Shakya Shrestha et al., 2016).

## 7 *Helicobacter pylori* biofilm and efflux pump promoted resistance

There is a widespread consensus that the development of antibacterial resistance in *H. pylori* is linked to the complex physiological process of biofilm formation (Tshibangu-Kabamba and Yamaoka, 2021). *H. pylori* strategically forms biofilms as a defense mechanism against external factors such as pH and oxidative stress. These biofilms predominantly consist of static cell clusters enveloped by an extracellular matrix composed of proteins and extracellular DNA (Guzman-Soto et al., 2021). Stark provided the first evidence of the ability of *H. pylori* to create biofilms under specific conditions, particularly in supplemented 10% *Brucella* broth (Stark et al., 1999). The process of *H. pylori* biofilm formation encompasses stages such as bacterial adhesion, biofilm assembly, maturation, and dispersion. Within mature biofilms, the majority of bacterial cells assume a spherical shape, considered the dormant state of *H. pylori*, contributing to its resistance and disease induction potential (Elshenawi et al., 2023). *H. pylori* biofilms exhibit significantly greater antibacterial tolerance than their planktonic counterparts. Notably, the amoxicillin tolerance level was shown to be up to 1,000 times greater, while that to clarithromycin, levofloxacin, and metronidazole was 31, 16, and 8 times greater, respectively, in biofilms (Fauzia et al., 2020). However, the mechanism by which biofilms enhance *H. pylori* tolerance remains a subject of ongoing research. This phenomenon may be associated with the upregulation of genes involved in modifying the cell wall of *H. pylori* within the biofilm, such as *UppS* (Hathroubi et al., 2020; Rahman et al., 2020) and *PdgA* (Zhao et al., 2023).

Efflux pumps are a type of multidrug transporters found in the bacterial cell membrane (Hou et al., 2022). These pumps actively transport antibacterial drugs out of bacterial cells, thereby reducing the drug concentration within the cells and promoting drug resistance. In the case of *H. pylori*, efflux pumps play a central role in the development of multidrug resistance and mediate resistance to antibacterial agents such as amoxicillin, metronidazole, clarithromycin, and tetracycline (Cai et al., 2020). In clinical multidrug-resistant *H. pylori* strains, the expression levels of efflux pump genes are significantly increased. These efflux pump-encoding genes are notably upregulated within biofilms, further enhancing the tolerance of *H. pylori* to antibiotics (Ge et al., 2018). Yonezawa et al. demonstrated that the expression of outer membrane proteins from family 5 efflux pumps, including *hefa* (*HP0605*), *hefd* (*HP0971*), and *hefG* (*HP1327*), was significantly greater in biofilm-forming cells than in planktonic cells (Yonezawa et al., 2019). These findings confirm that efflux pumps can collaborate with biofilms to amplify drug resistance and play a pivotal role in enabling *H. pylori* to thrive within biofilms, which are unaffected by antibiotics.

While *H. pylori* cells residing within biofilms may shield themselves from antibacterial drugs and the immune system, the precise impacts of *H. pylori* biofilms on antibiotic resistance and immune system clearance *in vivo* remain to be fully elucidated. Existing evidence indicates that *H. pylori* can indeed form biofilms, and it has been observed that genes related to efflux pumps are upregulated within biofilms. However, the mechanisms through which biofilms influence efflux pumps remain unclear. Future research aimed at enhancing the efficacy of eradication therapy should prioritize exploring the yet uncharted role of biofilms in *H. pylori* infections.

## 8 New approaches

**Figure 5:**
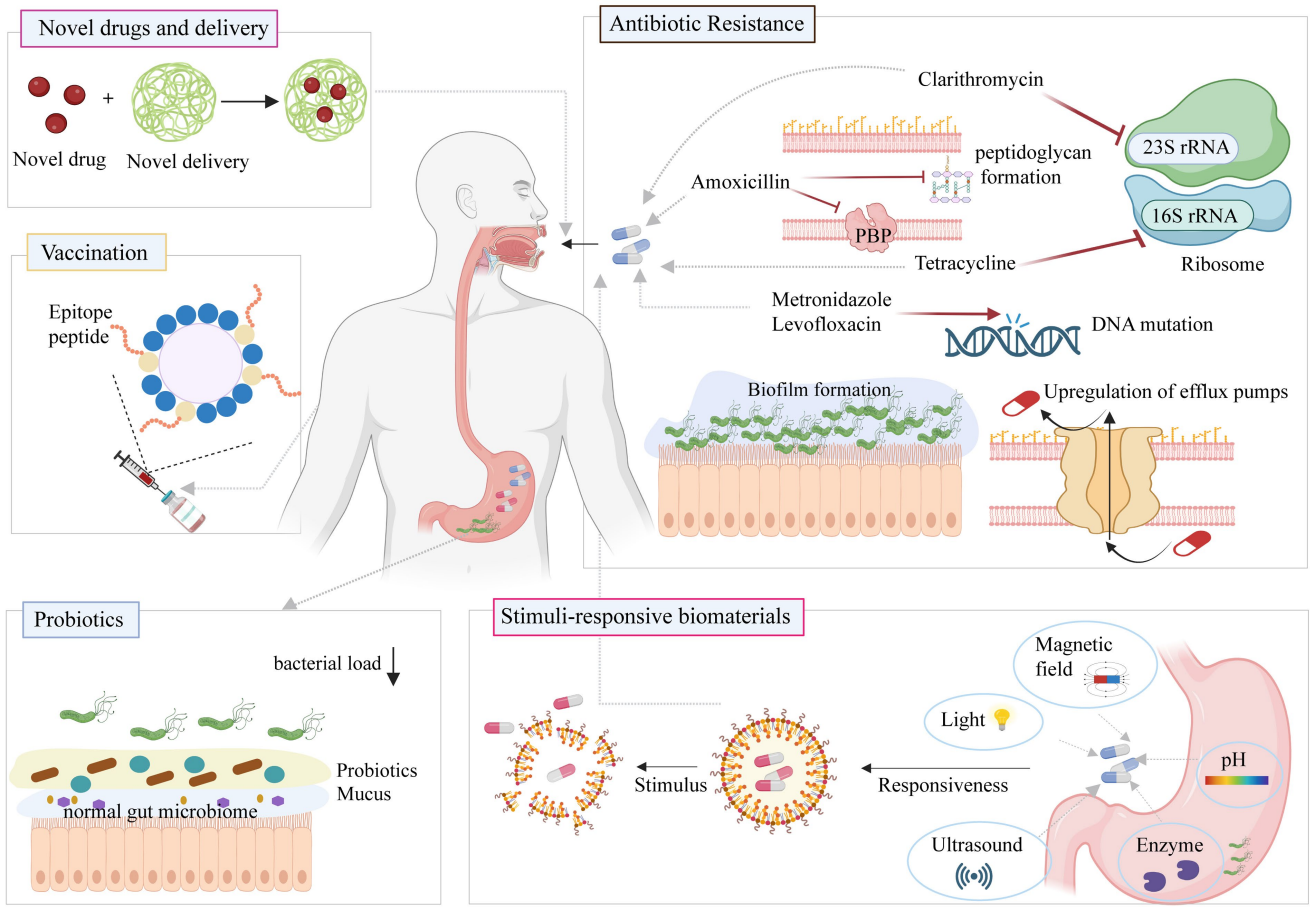
Classic and new approaches against *H. pylori* infection. Figure is created based on BioRender (BioRender.com).

Drug resistance of *H. pylori* has a significant impact on therapeutic efficacy. Although continuous improvements of antibacterial drug combinations can make them effective in the short term, the efficacy tends to decline over time. This fact emphasizes that solving the problems of drug resistance and *H. pylori* eradication may be difficult if antimicrobial drugs continue to be the focus. It may be necessary to change the strategy and find a different approach to address this issue (Figure 5).

### 8.1 Natural products

Eradication therapy for *H. pylori* remains a challenge. The development of new natural drugs continues to reduce side effects, improve patient compliance, and thus increase eradication rates (Hafeez et al., 2021). Natural products involving terpenoids, polyphenols, and alkaloids (Chen C. L. et al., 2023; Deng et al., 2024). Terpenoids like carvacrol disrupt bacterial membranes and reduce ATP synthesis, leading to bacterial death (Campestre et al., 2021). Polyphenols such as kaempferol inhibit reproductive enzymes, destabilize the cytoplasmic membrane, and regulate MAPK and NF-κB pathways to mitigate inflammatory damage (Bi et al., 2022). Alkaloids, such as coptisine, inhibit urease activity and disrupt cell membranes (Habimana et al., 2023). Combining plant foods with antibiotics enhances *H. pylori* eradication and offers potential for preventing associated gastric disorders (Liu et al., 2023).

Aminoglycosides such as gentamicin and netilmicin are currently considered new *H. pylori* eradication regimens. A gentamicin-intercalated montmorillonite hybrid remains in the gastric mucosal layer for up to 1 h, producing a local therapeutic effect against *H. pylori*, without side effects (Jeong et al., 2018). Animal experiments showed that topical aminoglycoside therapy with a montmorillonite delivery system was effective against *H. pylori* (Lee et al., 2019; Jeong et al., 2020). Tomokazu investigated the efficacy of intervenolin, a novel quinolone derivative, as a potential monotherapy for *H. pylori* infection. This study demonstrated that AS-1934 exhibited potent activity against various strains of *H. pylori in vitro*. In mice infected with *H. pylori* SS1, treatment with AS-1934 as a monotherapy resulted in a better eradication rate than treatment with triple therapy. Importantly, AS-1934 is stable at pH 2, but its activity could be blocked by a PPI (Ohishi et al., 2018).

### 8.2 Probiotic intervention

Compared with those in *H. pylori*-negative individuals, the main genera of gastric bacteria that were found to be abundant in *H. pylori*-positive individuals were *Streptococcus*, *Neisseria*, *Prevosia*, *Roche*, *Clostridium*, *Vermicelli*, and *Haemophilus* (Monstein et al., 2000; Bik et al., 2006). In addition to causing gastroduodenal disease, *H. pylori* infection may be involved in metabolic syndrome (Pellicano et al., 2020). Infection with *H. pylori* causes metabolic imbalances and disrupts host-gut microbiota interactions, leading to ecological dysregulation and triggering an inflammatory response (Behzadi et al., 2024). The metabolism of the intestinal microbiota is disrupted after *H. pylori* infection. The combination of *Lactobacillus rhamnosus* LGG-18 and *Lactobacillus salivarius* Chen-08 probiotics could reduce the severity of gastritis and inhibit precancerous lesions caused by *H. pylori* infection. However, it is clear that the supplementation with probiotics does not reduce the number of *H. pylori* cells (He et al., 2022a). After 14 days on bismuth-containing quadruple therapy combined with probiotics, 276 first-time *H. pylori*-positive patients had a lower incidence of gastrointestinal adverse events than did the placebo group (23.6% vs. 37.7%), but there was no significant difference in eradication rates (He et al., 2022b). The probiotic Miya-BM® was also used to assist in *H. pylori* eradication, and 468 *H. pylori*-positive patients were randomized to receive a PPI and amoxicillin or clarithromycin (PPI group); vonoprazan and amoxicillin or clarithromycin (vonoprazan group); or a PPI, amoxicillin or clarithromycin and the probiotic (probiotic group). The results showed no significant differences in adverse reactions among the three groups (Mukai et al., 2020). Fifty-six patients were randomly divided into quadruple therapy, probiotic supplement quadruple therapy, and probiotic monotherapy groups. The adjuvant probiotic therapy could partially help in the recovery of the gastric microbiota after *H. pylori* eradication; however, the results showed that the use of probiotics as a single eradication therapy not only had no benefit in patients infected with *H. pylori* but increased the possibility of potential infection with pathogenic bacteria such as *Clostridium* (Yuan et al., 2021). Several scholars have suggested combining inactive probiotics with triple therapy to eliminate *H. pylori*. To investigate the effectiveness of this approach, 200 untreated *H. pylori*-positive adult patients were randomly assigned to receive either inactive *L. reuteri* DSM 17648 (LR group) or a placebo for two weeks. After the initial two weeks, both groups underwent two weeks of triple therapy (esomeprazole, amoxicillin, and clarithromycin). The results showed that adding inactive *L. reuteri* DSM 17648 to triple therapy did not increase the eradication rate of *H. pylori*. Nevertheless, it helped establish a beneficial microbial spectrum and reduce side effects such as abdominal distension and diarrhea (Yang et al., 2021). Biofermin-R is a preparation of the multidrug-resistant lactic acid bacterium *Enterococcus faecium* 129 BIO 3B-R. When used in combination with vonoprazan, Biofermin-R can increase the eradication rate and help maintain species diversity in the intestinal microbiota (Kakiuchi et al., 2020). More research is needed to verify the effectiveness of this approach. Overall, the effectiveness of probiotic strains may depend not on their ability to colonize the gastrointestinal tract but rather on their ability to affect gene expression and metabolite levels and directly influence the intestinal barrier and immune cells. Although probiotics may play a role in eradicating *H. pylori*, further research is needed to confirm their effects.

### 8.3 Vaccination

Vaccination is a new strategy to address the rising problem of bacterial antibiotic resistance (Czinn and Blanchard, 2011; Dos Santos Viana et al., 2021). Vaccination is considered the best way to boost the production of secretory IgA (SIgA) in the stomach lining, which prevents bacterial colonization. *H. pylori* relies on virulence factors such as urease and heat shock protein A (HspA) to survive and colonize the stomach. Urease contains the UreA and UreB subunits, with UreB being a well-known antigen associated with *H. pylori*. Both subunits have been extensively studied for vaccines against *H. pylori* infection. Many promising *H. pylori* vaccine candidate antigens have been used, including UreB, VacA, CagA, HspA, neutrophil-activating protein (NAP), and rsLPS (Sutton and Boag, 2019; Yunle et al., 2024). Most of the vaccines are in the preclinical stage (Table 2). Paidamoyo modified *Bacillus subtilis* spores to present the protective antigens UreA and UreB from *H. pylori* on their surfaces. When these spore vaccines were orally administered to mice following *H. pylori* exposure, the *H. pylori* colony counts in the stomach decreased by approximately one log (Katsande et al., 2023). The immunogenicity and immunoprotection of the *Shigella* vector vaccine SH02 expressing an *H. pylori* UreB-HspA fusion protein were evaluated in a mouse model. The basic immunization consisted of either three oral administrations or two oral immunizations, followed by a third immunization involving a subcutaneous injection of rUreB-HspA for immune enhancement. Both approaches significantly reduced *H. pylori* colonization and gastric inflammation in mice (Zhang et al., 2022).

The EpiVax *H. pylori* vaccine, which is based on a multiepitope approach, is currently in the preclinical phase. This vaccine employs multiepitope DNA priming, followed by peptide boosting to enhance immunity. Notably, compared with that in mice that received either an empty plasmid or a complete *H. pylori* lysate intranasally as immunogens, *H. pylori* colonization was significantly reduced in mice that received this vaccine through intramuscular injection (Moss et al., 2011). Multivalent subunit vaccines that contain CagA, VacA, and NAP have proven effective in reducing *H. pylori* infections. Guo et al. actively contributed to the development of an *H. pylori* vaccine. They designed an immune adjuvant called NAP, along with three specific functional fragments (CagA 302–437, VacA 146, and VacA 332–494) derived from CagA and VacA. These components were incorporated into a multivalent epitope vaccine known as FVpE, which was derived from the urease multiepitope peptide fused with CTB. Oral therapeutic immunization with this vaccine reduced the numbers of *H. pylori* colonies in the stomachs of Mongolian gerbils (Gisbert and McNicholl, 2017; Guo et al., 2019).

Vaccines stimulating mucosal immune responses raise concerns when weakened viruses or pathogens are used because of the risk of virulence reversion. CD4^+^ T cells serve as crucial effector cells in the adaptive immune response against *H. pylori*. While this response is traditionally considered to be Th1-mediated, recent studies have introduced Th17 cell subsets into the context of *H. pylori* infections. Activation of both Th1 and Th17 cells leads to the production of cytokines such as IFN-γ, IL-17, and TNF-α, which collectively contribute to the defense against inflammatory *H. pylori* infections (Karkhah et al., 2019). Hence, their group enhanced the oral vaccine by creating a recombinant *Lactococcus lactis* vaccine, LL-plSAM-FVpE, which targets microfold cells more efficiently. The protective immunity provided by LL-plSAM-FVpE seems to be associated with specific sIgA and IgG antibodies, as well as with CD4^+^ T-cell responses against several important *H. pylori* virulence factors (such as urease, CagA, VacA, and NAP) (Guo L. et al., 2022). Further evaluation of this vaccine in animal models and clinical trials will be a research focus. Some scholars have suggested various methods, including targeting γ-glutamyl transpeptidase (Rossi et al., 2012), outer inflammatory protein A (Soudi et al., 2021), and *H. pylori* neutrophil-activating protein (Peng et al., 2018; Liu et al., 2020), for vaccine design. These studies need further experimental verification for the direct evaluation of *H. pylori* colonization and inflammation after treatment.

### 8.4 Stimulus-responsive biomaterials

Two main types of stimulus-responsive biological materials include those responsive to endogenous stimuli, such as pH and enzymes, and those responsive to exogenous stimuli, including magnetic fields, photodynamic forces, and ultrasound. An acidic gastric environment significantly influences the residence time and drug behavior of antibacterial agents. Jing et al. assessed pH-sensitive urea-conjugated chitosan/sodium tripolyphosphate nanoparticles specifically designed to combat *H. pylori* infections. The nanoparticles utilize urea as a targeted head group to enhance their affinity for *H. pylori*. Upon encapsulating amoxicillin at a concentration of 0.5 μg/mL within the nanoparticles and incubating them for 24 h, the inhibition rate against *H. pylori* exceeded 80%. However, further comprehensive *in vivo* experiments are necessary to validate the efficacy of these nanoparticles in eradicating *H. pylori* (Jing et al., 2016). Yang et al. employed magnetic field stimulation to prolong the gastric residence time of novel nanocarriers loaded with amoxicillin. These nanocarriers, composed of chitosan and polyacrylic acid, were specifically designed to deliver superparamagnetic iron oxide in response to an externally applied magnetic field. The rate of *H. pylori* eradication following oral administration of amoxicillin (33 mg/kg)-loaded nanoparticles, combined with 30 min of daily magnetic field treatment over a 7-day period reached 60%(Yang et al., 2020).

A cascade of potent oxidative molecules, including superoxide anions (O^2−^) and hydroxyl radicals (•OH), generated by exogenous reactive oxygen species (ROS), has the capacity to inflict damage upon bacterial lipids, proteins, and DNA, ultimately resulting in bacterial death (Hong et al., 2019). Yu et al. created a novel pH-responsive ROS nanogenerator, termed Fe-HMME@DHA@MPN. It comprises a metal–organic nanostructure (Fe-HMME) formed by coordinating the sonosensitizer hematoporphyrin monomethyl ether (HMME) with ferric iron. When loaded with dihydroartemisinin (DHA) as a hydroperoxidant, the nanogenerator has an acidic pH-sensitive metal shell. Following oral administration, it rapidly dissociates in the stomach and is activated by ultrasound. Then, DHA, which serves as a ROS source, is released through the Fenton reaction, generating •OH to kill *H. pylori* (Yu et al., 2023).

Zhou et al. developed a novel nanoparticle system capable of ultrasonically driven motion and ultrasonically excited afterglow emission (Zhou et al., 2022). Specifically, an enzyme-responsive polydopamine layer was applied to encapsulate the clarithromycin and luminescent particle complex, followed by fluorescence quenching. Upon oral administration, ultrasound activation of the nanoparticles facilitates their movement through mucus to the site of *H. pylori* infection. Bacterial secretion of phospholipase triggers the dissociation of responsive polydopamine layer, thereby releasing the encapsulated clarithromycin to eradicate the infection. *In vivo* experiments demonstrated that nanoparticle-mediated delivery of clarithromycin significantly reduced the bacterial burden at lower antibiotic doses, without causing notable surface degradation or erosion in the gastrointestinal tract.

### 8.5 Phage therapy

Phage therapy is a method of treating bacterial infections by employing phages, which are viruses that infect and reproduce within bacteria (Kushwaha et al., 2024). Once a bacterium is infected, the phage utilizes the bacterium’s metabolic machinery to replicate, ultimately leading to the bacterium’s lysis and the release of more phages, thereby exerting an anti-infective effect. There are two primary types of phages: virulent phages and temperate phages (Hatfull et al., 2022). Virulent phages infect the host bacteria rapidly, leading to bacterial lysis and death, making them suitable for rapidly clearing bacterial infections (Jonczyk-Matysiak et al., 2019). In contrast, temperate phages replicate more slowly and cause gentler bacterial killing, making them preferable for chronic bacterial infections with less inflammatory reaction and tissue damage (Hatfull et al., 2022).

In approximately 20% of *H. pylori* isolates, prophages like PhiHp33 from strain B45 are present (Munoz et al., 2020). However, the precise function of prophages remains unclear, and their link to disease has been scarcely explored. In 2008, a 100 nm-sized phage was isolated from human feces but wasn’t further investigated (Munoz et al., 2020). In 2013, two lysogenic phages (ΦHPE1 and ΦHPE2) were identified in wastewater, belonging to the Podoviridae and Siphoviridae families, yet their antimicrobial potential was not evaluated (Abdel-Haliem and Askora, 2013). Recently, *H. pylori* (Hp φ) was isolated from gastric biopsies-bound lactoferrin and adsorbed on hydroxyapatite nanoparticles, which effectively reduced bacterial colonization and inflammatory damage (Cuomo et al., 2020).

Phages show promise as a therapeutic avenue for *H. pylori*, yet the field remains in its early stages, with the phage genome sequence yet to be determined. Moving forward, efforts should be directed toward identifying and designing phages specifically targeting *H. pylori*.

### 8.6 Antimicrobial peptides

Due to the escalating issue of antibiotic resistance, antimicrobial peptides are emerging as an alternative therapy to antibiotics for combating bacterial infections. These peptides, known as natural antimicrobial peptides (AMPs), are innate immune molecules produced in nearly all organisms (Kumar et al., 2018). Predominantly of eukaryotic origin, AMPs exhibit direct bactericidal activity. Typically composed of 10–100 amino acids, AMPs are characterized as cationic or amphoteric (Mohaideen et al., 2023). They act by interacting with the negatively charged head groups of phospholipids on the cell membranes of both Gram-positive and Gram-negative microorganisms through electrostatic forces. This interaction results in the formation of pores or channels, disrupting the cell membrane and ultimately leading to cell lysis. Additionally, the phospholipids of Gram-positive microorganisms and the lipopolysaccharides of Gram-negative bacteria continuously provide negative charges to the bacterial surface, thereby further enhancing the interaction with AMPs (Mahlapuu et al., 2020).

Natural antimicrobial peptides exhibit instability in the gastrointestinal tract, poor absorption, distribution, and rapid excretion, leading to low bioavailability (Kumar et al., 2018). Xiong et al. synthesized pH-sensitive antimicrobial peptides with a transformable helix–helix conformation, demonstrating low toxicity at pH 6–8 and potent antimicrobial activity against *H. pylori SS1* and drug-resistant strains at pH 2. Orally administered to C57BL/6 J mice, these peptides achieved a comparable eradication rate of *H. pylori SS1* to the positive control triple drugs, with reduced side effects on intestinal bacteria.(Xiong et al., 2017). Fonseca et al. developed a novel microfluidic system for AMP-nanoparticles that is stable and exhibits rapid bactericidal activity in acidic environments. This microfluidic device is simple, quick, minimally toxic to cells, and shows great potential for *in vivo* experiments to validate its effectiveness in eradicating *H. pylori* intragastric colonization (Fonseca et al., 2024). Zhang et al. designed genetically encoded *H. pylori*-responsive bactericidal protein crystals to improve the bioavailability of antimicrobial peptide (LL-37) in the stomach. Oral administration of the LL-37 significantly decreased gastric colonization of *H. pylori* in mice, mitigated inflammatory injury, and had no impact on the intestinal microbiota (Zhang W. et al., 2023).

Antimicrobial peptides hold promise for treating infections, but face challenges in clinical application. The stomach’s acidity and GI tract enzymes degrade orally administered peptides. Further research is needed to understand their mechanisms, improve chemical properties, enhance stability, and reduce manufacturing costs. Increased investment in AMP-based drug development is necessary to advance medical practice.

### 8.7 Alteration of microaerobic environment

*H. pylori* has strict oxygen requirements. The optimal oxygen concentration range in air is 5 to 8% (Dai et al., 2022), which is equal to 1.7 to 2.0 mg/L in aqueous solutions at 37°C. When the oxygen concentration in the environment is outside this range, *H. pylori* cannot survive. We carried out a series of experiments that showed a good eradication effect of altering the oxygen concentration on *H. pylori* (Di et al., 2020). The addition of 0.02 to 2.0 mg/mL Hydrogen peroxide increased the oxygen content in an aqueous solution (or rabbit gastric fluid) 1.2 to 3.9 (4.2 to 5.2) times, from 6.7 (3.6) μg/mL to between 7.8 and 26.0 (15.2 to 18.7) g/mL, respectively. In Columbia blood agar with *H. pylori* special peptone, hydrogen peroxide significantly inhibited the growth of *H. pylori*. The inhibition potency of hydrogen peroxide was greater than that of colloidal bismuth subcitrate. Furthermore, similar to other antibacterial drugs, colloidal bismuth subcitrate easily promoted the development of resistant strains of *H. pylori*, which sharply increased the MIC and minimum bactericidal concentration of the antibacterial drug. Hydrogen peroxide did not easily promote resistance of *H. pylori*. *In vivo* experimental results showed no *H. pylori* colonization in the stomachs of Mongolian gerbils in the hydrogen peroxide group, suggesting that the killing effect of hydrogen peroxide on *H. pylori* was greater than that of triple drugs (Di et al., 2020). A low concentration of hydrogen peroxide does not cause gastric injury or inflammatory reactions and has no significant toxicity.

Donelli et al. (1998) confirmed that when the oxygen concentration was reduced to 4%, cultured *H. pylori* could still reproduce, and the number of bacterial cells increased with time. However, when the oxygen concentration was reduced to 2–3%, *H. pylori* did not reproduce, and the number of bacterial cells decreased with time. Moreover, when the oxygen concentration was reduced to 0–1%, the number of bacterial cells decreased by 90% after 24 h of culture, and all cells died within 7 days. Using this strategy, we recently reduced the oxygen level in water by adding vitamin C (a reducing agent) to reduce the oxygen concentration in gastric juice and showed that this resulted in an anti-*H. pylori* effect. It was reported that vitamin C could increase the clearance rate of *H. pylori* after antibacterial treatment (Hussain et al., 2018; Cai et al., 2021), which supports our results, but the effect was still unsatisfactory. If strongly reducing drugs are used to consume oxygen in gastric juice so that the oxygen concentration is greatly reduced or oxygen is eliminated, *H. pylori* will inevitably fail to survive, and its eradication will be achieved. Sodium sulfite is a potent reducing agent that reacts with dissolved oxygen in aqueous solutions, effectively depleting oxygen. It is a food additive that restricts bacterial contamination and can be safely consumed at concentrations up to 5000 ppm (Irwin et al., 2017). Our latest results showed that 1, 3, and 10 mg/mL sodium sulfite lowered the concentration of oxygen in rabbit gastric juice from 2 to 0.2 mg/L. Sodium sulfite significantly inhibited the growth of *H. pylori* (ATCC 43504 and SS1 strains) in both solid and liquid cultures *in vitro*. Subsequently, we used the Mongolian gerbil infection model and confirmed the superior efficacy of sodium sulfite in eradicating *H. pylori*. Additionally, in mice, we showed a lower recurrence rate with sodium sulfite than with triple control drugs following the eradication of *H. pylori*. We also conducted acute and long-term toxicity experiments, which revealed that the LD50 of sodium sulfite in Kunming mice was 6.8 g/kg. After 6 weeks of continuous administration to rats, no significant differences in blood biochemical indicators, the body weight, or food intake were detected between the 0.3 g/kg sodium sulfite group and the control group (Huang et al., 2023).

The Hcp protein is a cysteine-rich *Helicobacter* protein. *HcpE* contains 19 cysteines and is the most prominent member of the Hcp family (Dumrese et al., 2009). Mittl et al. verified an *HcpE* IgG antibody in patients with *H. pylori* infection and confirmed that the Hcp protein was specific to *H. pylori*, which makes it a potential therapeutic target (Mittl et al., 2003). Lester et al. demonstrated that *HcpE* acted as a substrate for a dimeric oxidoreductase (*HP0231*), which transfers electrons to the respiratory chain for reoxidation in the *H. pylori* oxidation pathway (Grzeszczuk et al., 2018). Knocking out *HP0231* resulted in a decreased ability of *H. pylori* to maintain redox homeostasis (Lester et al., 2015). *H. pylori* adjusts its metabolism in response to hypoxic conditions. Under normal oxygen levels, it relies on microaerobic respiration for energy production (Kelly et al., 2001). In a hypoxic state, microaerophilic respiration is blocked in *H. pylori*, resulting in a sharp decrease in the ATP content and significant downregulation of the HcpE protein and antioxidant/reducing steady-state enzymes (catalase and superoxide dismutase). This leads to an increase in the ROS content, damaging the bacterial structure and eventually causing bacterial death due to energy exhaustion and structural destruction. Thus, regulating the oxygen environment for *H. pylori* growth may have antibacterial effects, with HcpE identified as a potential target (Huang et al., 2024).

Overall, altering the oxygen concentration in the gastric solution is an innovative and intriguing approach for eradicating *H. pylori*. This has shifted our focus away from antimicrobials and combinations of drugs. This concept may serve as a valuable source of inspiration for future *H. pylori* treatments.

### 8.8 Agonists and antagonists in anti-*H. pylori* infection

In addition to devising novel strategies for directly eradicating *H. pylori*, researches have also been toward counteracting the pathogenesis of *H. pylori* infection. When persistently infected with *H. pylori*, gastric mucosal epithelial cells (GES-1) exhibit reduced sensitivity of Toll-like receptor (TLR) 6 to *H. pylori* components, resulting in decreased expression of IL-1β and IL-8. Treatment with Pam2CSK4, a TLR6 agonist, restores TLR6 expression and reduces *H. pylori* colonization in gerbil gastric mucosa (Zhang et al., 2024). SHP1, the only homolog of the tyrosine phosphatase SHP2 expressed in gastric epithelial cells, negatively regulates the *H. pylori* virulence factor CagA (Saju et al., 2016). It inhibits *H. pylori*-induced migration and invasion of GES-1 and gastric cancer cells. Sorafenib, acting as an SHP1 agonist, inhibits *H. pylori*-induced proliferation and metastasis (Chen S. et al., 2024). A2BR, a functional protein in gastric mucosal wall cells, activates the p38MAPK pathway and exacerbates oxidative stress. Treatment with BAY60-6583 (an A2BR agonist) exacerbates gastric ulceration in rats, while PSB1115 (an A2BR antagonist) has the opposite effect. Incubation of GES-1 cells with agonists increased apoptosis, migration, and oxidative stress, effects that are reversed by inhibitors of the p38MAPK pathway (Tang et al., 2023). In summary, combating *H. pylori* infection can be approached from various angles, and agents such as A2BR antagonists, TLR6 agonists, and SHP1 agonists hold promise as novel therapeutic strategies against *H. pylori* infection in the future.

## 9 Conclusions and future perspectives

Antibacterial drugs show great therapeutic effects against *H. pylori* infection, but sometimes, they are not sufficient to eradicate *H. pylori*. In the past decade, approximately 10–30% of all first-and second-line eradication therapies recommended by global treatment guidelines have failed, which led to the development of triple therapy. Quadruple therapy, which involves adding another antimicrobial or replacing it with an acid inhibitor, was developed to overcome this problem. The wide range of antimicrobial agents extensively used for the eradication of *H. pylori* has led to an increase in drug resistance over time (Tshibangu-Kabamba and Yamaoka, 2021). As a result, retreatment and subsequent cure have become more difficult (Thung et al., 2016; Malfertheiner et al., 2017). To address this issue, new drugs and treatment strategies are necessary.

Notwithstanding, further study of the mechanisms of drug resistance in *H. pylori* is essential. Whole-genome sequencing predicted resistance in *H. pylori* and showed that antimicrobial resistance is primarily due to point mutations in the genome. For example, clarithromycin resistance in *H. pylori* is strongly associated with the presence of the A2146C, A2146G, or A2147G mutations in exon V of the 23S rRNA gene (Lauener et al., 2019). Mutations G94E in the *pbp1A* gene, C2173T and G2212A in the 23S rRNA gene, T239M in the *gyrA* gene, G122R in the *rdxA* gene, and A70T and A138V in the *frxA* gene have been found only in drug-resistant strains (Domanovich-Asor et al., 2020). The *pbp1A* gene mutation is a major factor causing amoxicillin resistance, while levofloxacin resistance may be mainly attributed to mutations in single or dual genes encoding DNA gyrase subunits A and B (*gyrA* and *gyrB*) in the quinolone resistance-determining region (QRDR). Mutations at codons 87 (N to K, I, or T) and 91 (D to Y, G, or H) in the QRDR of *gyrA* were found in 91% of resistant strains (Tshibangu-Kabamba et al., 2020). Although these studies have some value, it should be noted that gene mutations in *H. pylori* are not fixed in patients from different regions and some patients may have dual or multiple resistance. Therefore, the whole-genome sequencing results may not provide a representative conclusion.

Furthermore, new therapeutic strategies can be developed. In addition to traditional combination therapies, such as concomitant and sequential therapies, probiotics and vaccines have currently become popular new therapies. Probiotics can inhibit *H. pylori* colonization and produce antibacterial substances (Song et al., 2018). The eradication rate of *H. pylori* by using probiotics combined with the triple regimen was lower than that of the bismuth quadruple regimen, indicating that bismuth is irreplaceable and can inhibit the growth of probiotics. The acidic environment of the stomach may also affect the effect of probiotics. The eradication effects of different probiotics vary with treatment duration. Therefore, more detailed studies are needed on the use of probiotics as adjuvant therapy. An *H. pylori* vaccine was shown to be effective at preventing *H. pylori* infection (Zeng et al., 2015). However, one of the concerns regarding the treatment of *H. pylori* infection is whether the antibodies produced in the body can travel smoothly into the gut and not be destroyed by gastric acid or pepsin. Further evaluation is needed to confirm the long-term effectiveness of vaccines. Research on stimuli-responsive materials has shown promising potential against *H. pylori*; yet this field remains in its nascent stage. Responsive biomaterials pose challenges, including their complex compositions, intricate manufacturing processes, elevated costs, and complex treatment procedures. Future investigations should prioritize comprehensive *in vivo* verification of *H. pylori* clearance and further enhancement of biomaterial properties. Phage and antimicrobial peptide therapies are emerging for *H. pylori* eradication but are still in the early stages of development. Their antimicrobial mechanisms and effectiveness compared to first-line drugs require further investigation through *in vivo* experiments and preclinical validation.

**Figure 6:**
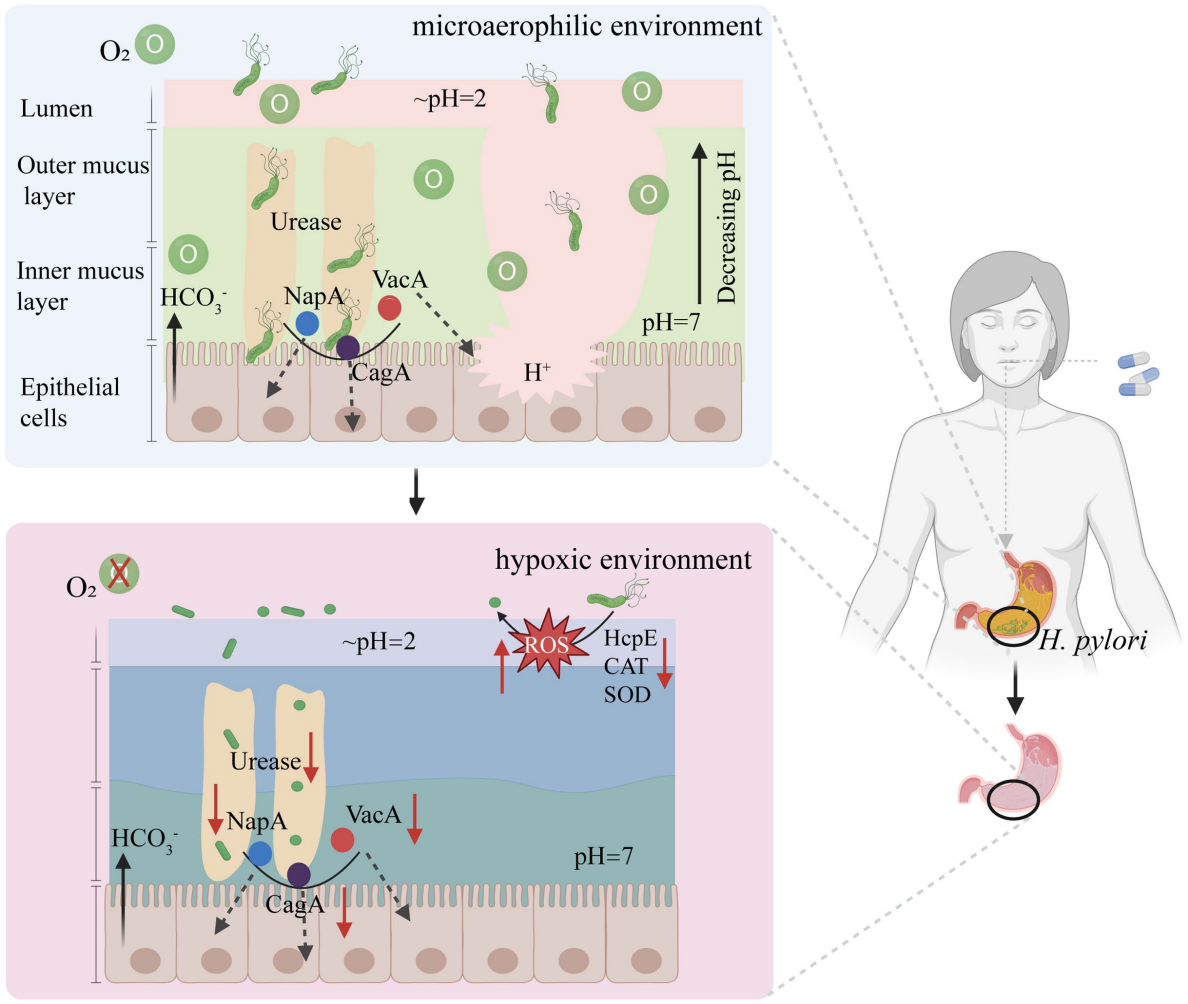
A new approach to eradicate *H. pylori* based on reducing intragastric oxygen concentration. HcpE, Helicobacter cysteine-rich protein; VacA, vacuolating toxin A; CagA, Cytotoxin-associated-gene A; NapA, neutrophil-activating protein A subunit; CAT, Catalase; SOD, Superoxide dismutase. Figure is created based on BioRender (BioRender.com).

A different path may lead to better results. Because a strictly microaerophilic environment is suitable for survival of *H. pylori*, a completely novel way to change the oxygen concentration that is required for the growth of *H. pylori* to a level at which *H. pylori* cannot survive (Di et al., 2020). has been proposed. Under normal microaerobic conditions in the stomach, *H. pylori* infection prompts the bacterium to employ various survival mechanisms. Upon reaching the mucus layer, urease aids in resisting the acidic gastric environment, while flagella facilitate movement toward mucosal epithelial cells for colonization and the release of pathogenic factors (such as NapA, CagA, and VacA) (Figure 6). However, in the hypoxic stomach environment, bacterial cells outside the mucus layer struggle to respire and metabolize because of limited oxygen availability. This leads to a sharp reduction in energy levels, causing a transition from the active rod-shaped forms to globular shapes, which is indicative of the dormancy status or eventual death. With diminished flagella and urease activities, bacterial cells are unable to penetrate the mucus layer and to colonize epithelial cells (Figure 6). The proposed method can efficiently eradicate *H. pylori* without generating drug resistance. This kind of clearance does not require drugs to enter cells and is easy to achieve. This strategy may be a good therapeutic option for eradicating *H. pylori* in the future.

## Author contributions

T-TH: Data curation, Formal analysis, Investigation, Methodology, Software, Visualization, Writing – original draft, Writing – review & editing. Y-XC: Conceptualization, Project administration, Supervision, Validation, Writing – original draft, Writing – review & editing. LC: Conceptualization, Data curation, Formal analysis, Investigation, Project administration, Resources, Software, Supervision, Writing – original draft, Writing – review & editing.

## Glossary

*H. pylori*: *Helicobacter pylori*
STT: Standard triple therapy
BQT: Bismuth-containing quadruple therapy
mBQT: Improved bismuth-containing quadruple therapy
CI: Confidence interval
PPI: Proton pump inhibitors
BabA: Blood group antigen-binding adhesion
SabA: Salivary acid-binding adhesion
*CagA*: Cytotoxin-associated gene A
*VacA*: Vacuolating cytotoxin A
*DupA*: Duodenal ulcer-promoting gene A
T4SS: Type IV secretion system
NAP: Neutrophil-activating protein
HDDT: High-dose dual therapy
MIC: Minimum inhibitory concentration
ROS: Reactive oxygen species
HspA: Heat shock protein A
*HcpE*: Cysteine-rich *Helicobacter* protein
GES-1: Gastric mucosal epithelial cells
TLR6: Toll-like receptor 6
